# A Generic Mechanism for Enhanced Cytokine Signaling *via* Cytokine-Neutralizing Antibodies

**DOI:** 10.1371/journal.pone.0149154

**Published:** 2016-02-12

**Authors:** Boris Shulgin, Gabriel Helmlinger, Yuri Kosinsky

**Affiliations:** 1 M&S Decisions LLC, Moscow, Russia; 2 Quantitative Clinical Pharmacology, Early Clinical Development, Innovative Medicines, AstraZeneca Pharmaceuticals LP, Waltham, Massachusetts, United States of America; Institut Pasteur, FRANCE

## Abstract

Enhancement or inhibition of cytokine signaling and corresponding immune cells responses are critical factors in various disease treatments. Cytokine signaling may be inhibited by cytokine-neutralizing antibodies (CNAs), which prevents further activation of cytokine receptors. However, CNAs may result in enhanced—instead of inhibitory—cytokine signaling (an “agonistic effect”) in various *in vitro* and *in vivo* experiments. This may lead to lack of efficacy or adverse events for cytokine-inhibiting based medicines. Alternatively, cytokine-antibody complexes may produce stronger signaling *vs*. cytokine alone, thereby increasing the efficacy of stimulating cytokine-based drugs, at equal or lower cytokine doses. In this paper, the effect of cytokine signaling enhancement by a CNA was studied in a generic mathematical model of interleukin-4 (IL-4) driven T-cell proliferation. The occurrence of the agonistic effect depends upon the antibody-to-cytokine binding affinity and initial concentrations of antibody and cytokine. Model predictions were in agreement with experimental studies. When the cytokine receptor consists of multiple subunits with substantially differing affinities (*e*.*g*., IL-4 case), the choice of the receptor chain to be blocked by the antibody is critical, for the agonistic effect to appear. We propose a generic mechanism for the effect: initially, binding of the CNA to the cytokine reduces free cytokine concentration; yet, cytokine molecules bound within the cytokine-CNA complex—and released later and over time—are “rescued” from earlier clearance *via* cellular internalization. Hence, although free cytokine-dependent signalling may be less potent initially, it will also be more sustained over time; and given non-linear dynamics, it will lead ultimately to larger cellular effector responses, *vs*. the same amount of free cytokine in the absence of CNA. We suggest that the proposed mechanism is a generic property of {cytokine, CNA, receptor} triads, both *in vitro* and *in vivo*, and can occur in a predictable fashion for a variety of cytokines of the immune system.

## Introduction

Cytokines are signaling molecules which regulate immune cells in terms of their proliferation, differentiation, activation and homeostasis, *via* interactions with immune cell surface receptors. Cytokines such as interleukins (ILs), interferons (IFNs), tumor necrosis factors (TNFs), colony-stimulating factors (CSFs), and monocyte chemoattractant proteins (MCPs) bind specific receptors, causing activation or inhibition of intracellular signaling pathways. Modulating cytokine signaling may causally and favorably affect drug therapeutic responses, in specific disease context. Cytokine-binding proteins such as cytokine-specific neutralizing antibodies (CNAs) or soluble receptors may be used when cytokine inhibition is required, for instance in drug therapeutic interventions against inflammatory diseases and symptoms. In such situations, a cytokine-binding protein would normally inhibit signaling by means of binding to the soluble cytokine, thereby preventing further binding of the cytokine to its cell-surface receptor(s).

This intuitive view has been challenged, however, by experimental observations which point to a rather paradoxical behavior, whereby CNAs (as well as soluble receptors), meant to inhibit cytokine signaling, did in fact promote and enhance the signaling effect, resulting for example in enhanced cell proliferation rather than proliferation inhibition (referred to, in this paper, as the “agonistic effect”). Such observations have been made for a variety of cytokines, including IL-2, IL-3, IL-4, IL-5, IL-6, IL-7, IL-15, TNF, G-CSF, in *in vitro* as well as *in vivo* studies [1–12]. Table 1 provides an overview, with context, of such paradoxical agonistic effects.

**Table 1:** Experimentally observed enhancement of cytokine signaling by cytokine-neutralizing antibodies (CNAs).

Cytokine	Cell type(s) affected by cytokine	Agonistic effect of CNA	Selected publications
IL-2	CD8+, CD4+ T cells, *in vivo*	T-cell proliferation is increased by anti-IL-2 mAb or by anti-IL-2 mAb/IL-2 complexes (stronger effect) at medium mAb concentrations	[7,8,10]
	CD8+ T cells, DC, NK cells, *in vivo*	CD8+ T cells, dendritic cells (DC), NK cells populations are increased by complex of IL2/antil-IL2 mAb. Anti- tumor effect observed	[13]
IL-3	Mucosal mast cells, *in vivo*	Increase in mucosal mast cells by IL-3/anti-IL-3 mAb complexes	[3,4]
IL-4	B cells, *in vivo*	Production of IgE by B cells is increased by IL-4/anti-IL4 mAb or by IL-4/sIL-4R at medium mAb concentrations	[1]
	Splenocytes, *in vivo*	Increase in splenocyte Ia expression by IL-4/anti-IL-4 mAb complexes	[3]
	CD45R0+ T cells, *in vitro*	Production of IFN-γ by T cells is increased by IL-4/sIL-4R at medium concentrations	[5]
	CD8+ T cells, *in vivo*	mIL-4/mAb complexes induce T-cell proliferation	[8]
IL-5	Bone marrow FDC-P1-CA1 cells sensitive to IL-5, *in vitro*	Cell proliferation increased when IL-5 is complexed with anti-IL-5 mAb at medium concentrations	[14]
IL-6	B9 hybridoma cells, *in vivo* and *in vitro*	Anti-IL-6 mAb enhances the ability of IL-6 to elicit hepatocyte effects (stimulation of fibrinogen levels), at medium anti-IL-6 concentrations.	[2,9]
IL-7	CD8+ T cells, *in vivo*	IL-7/anti-IL-7 mAb complexes increase IL-7 potency of T-cell proliferation	[11]
	CD8+ and CD4+ T cells, premature B-cells, *in vivo*	IL-7/anti-IL-7 mAb complex shows higher activity as compared to free IL-7	[15]
IL-15	CD8+ cells, NK cells, *in vivo* and *in vitro*	IL-15/sIL-15R complexes induce strong expansion of memory CD8 and NK cells	[16]
	CD8+, CD44_high, NK cells, *in vivo* and *in vitro*	IL-15R-Fc enhances the IL-15 potency to expand populations of NK and CD8+/CD44_high T cells	[17]
TNF	WEHI-164 and Meth A fibro-sarcoma, *in vivo*	TNFα/anti-TNFα-mAb complexes result in a 5- to 10-fold higher anti-tumor activity *vs*. free hTNF	[18]
G-CSF	CD11b+ Gr-1+ myeloid cells, *in vivo*	G-CSF/anti-G-CSF mAb complexes induce a 100-fold higher expansion of myeloid cells *vs*. G-CSF	[12]

If not taken into account and within the appropriate pathophysiological context, this paradoxical agonistic behavior may act unfavorably against antibody-based therapies of disease and/or contribute towards adverse events in patients. Alternatively, and again depending on context, CNA complexes may be exploited to produce stronger signaling *vs*. the corresponding cytokine alone, thereby increasing the efficacy of an antibody-based medicine at equal or lower administered dose(s) [1]. IL-2/anti-IL-2 antibody immune complexes, as well as complexes involving other cytokines, have been considered for treatment in immuno-oncology, auto-immune disorders, viral and bacterial infections, and multiple sclerosis [13,19–24].

Although various experimental and phenomenological studies of the agonistic effect have been reported, there still is a lack of understanding of the underlying mechanisms, including their quantitative and kinetic signatures. The proposed explanations of the phenomenon differ for different cytokines and are more of a hypothetical nature. As summarized by Létourneau *et al*. [10], several suggestions on a potential mechanism that underlies the agonistic effect have been made, yet “the mechanism still remains elusive”. In another publication [11], explanations for the underlying mechanism were put forward, yet with a conclusion that “the mechanism still remains enigmatic”.

In this paper, we explore this paradoxical, CNA-dependent agonistic effect in kinetic, quantitative, context-dependent terms, through a mathematical model that explores the phenomenon explicitly. Mathematical modeling provides an opportunity for an in-depth study of all plausible mechanisms underlying a system’s behavior, using currently available data for model building and qualification; it then also allows, *via* simulations, for the investigation of extrapolative scenarios which go beyond currently tested experimental conditions.

The mathematical model was developed for a particular case of T-cell proliferation activated by the cytokine IL-4; this model can be easily adapted to describe other members of the common γ-chain interleukin family, as well as for other cytokines.

We show that the CNA-dependent agonistic effect originates from basic biochemical properties of the cytokine-receptor system. It may, therefore, not be an isolated or unique phenomenon, as indeed observed experimentally for an increasing number of cytokine-antibody systems, both *in vitro* and *in vivo* (Table 1) (see also the review [9]).

## Materials and Methods

### Cytokine-receptor interactions; Overview of base model

**Fig 1:**
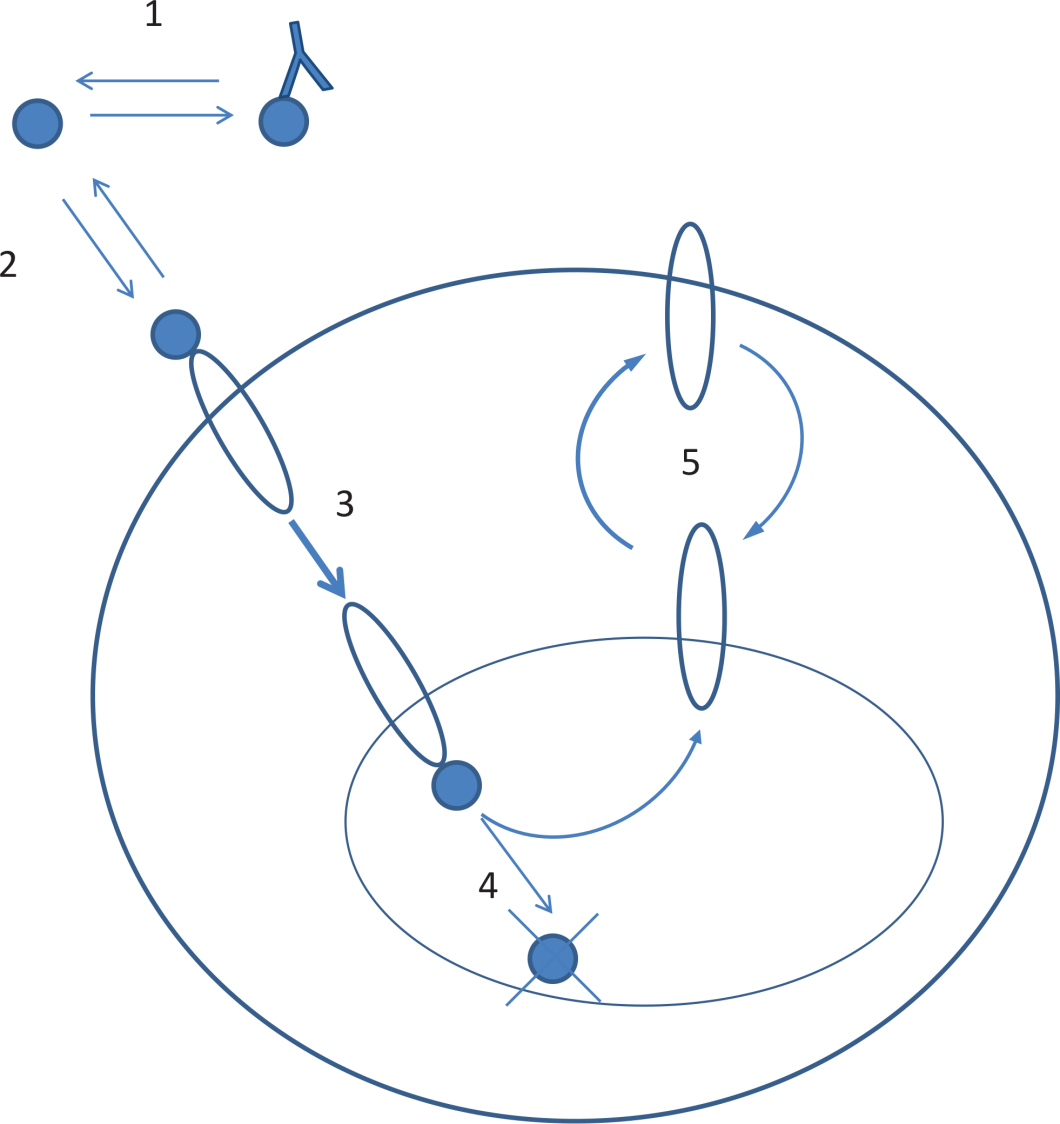
Schematics of main biochemical reactions in a cytokine-receptor model. 1 –binding of cytokine with antibody, 2 –binding of cytokine with receptor on the cell membrane, 3 –internalization of cytokine/receptor complex (relatively fast), 4 –degradation of cytokine in endosome (lysosome), 5 –recycling of receptor from endosome to the cell surface and internalization (relatively slow) of free receptor.

Our model describes an *in vitro* system, which includes immune cells such as specific B- or T-lymphocytes with their cell surface-bound cytokine receptors, as well as soluble cytokines and their corresponding CNAs. Cytokine/receptor interactions usually differ in quantities and kinetics, given the different cytokines and receptors involved; such differences may be captured using different values for the key parameters described in the model, while the model structure itself may be unchanged, for the various cytokine/receptor systems under study (Fig 1).

In the proposed model, free cytokine may bind to its cell membrane-bound receptor, thereby forming a cytokine/receptor signaling complex. The antibody may reversibly bind to the cytokine, thereby preventing cytokine binding to the membrane receptor (or to specific receptor subunit(s)–discussed subsequently). In some cytokine/receptor systems, free cytokine may bind to multiple subunits of the same receptor; this has been also modeled previously, via binding to a single receptor unit and without consideration of binding to distinct subunits, for example in the cases of IL-2 [25], IL-7 [26], and IL-15 [27]. This simplified approach, however, would not adequately describe potentially important effects, whereby specific antibodies preferentially block binding to specific subunits of a given receptor–a feature which is important to consider in the present modeling study.

The formed cytokine/receptor signaling complex may subsequently be internalized, with further degradation of cytokine molecules in the lysosome, followed by recycling of receptor subunits to the cell surface. In relevant cases (e.g., interleukins with a common γ-chain), degradation and synthesis of receptor subunits could also be included in the model. Typically, cytokine binding strongly accelerates the internalization process of complexed receptor subunits, as compared to the free, unbound receptor subunits. The relatively fast internalization of the signaling complex, followed by cytokine degradation, plays an important role as a limiting mechanism in the overall signaling pathway.

### T-cell activation by IL-4; Development of a mechanism-based model

IL-4 may bind *via* two distinct epitopes to its hetero-dimeric receptor, which is composed of the IL-4Rα subunit and the common γ-chain (γ_c_). Spatial modeling of this triangular complex [28] showed that certain IL-4-neutralizing monoclonal antibodies (mAb) may block only one of the two epitopes at the cytokine’s molecule surface. The present study investigates and predicts subsequent biological effects of a mAb blocking IL-4 binding to either IL-4Rα only, or to γ_c_ only.

**Fig 2:**
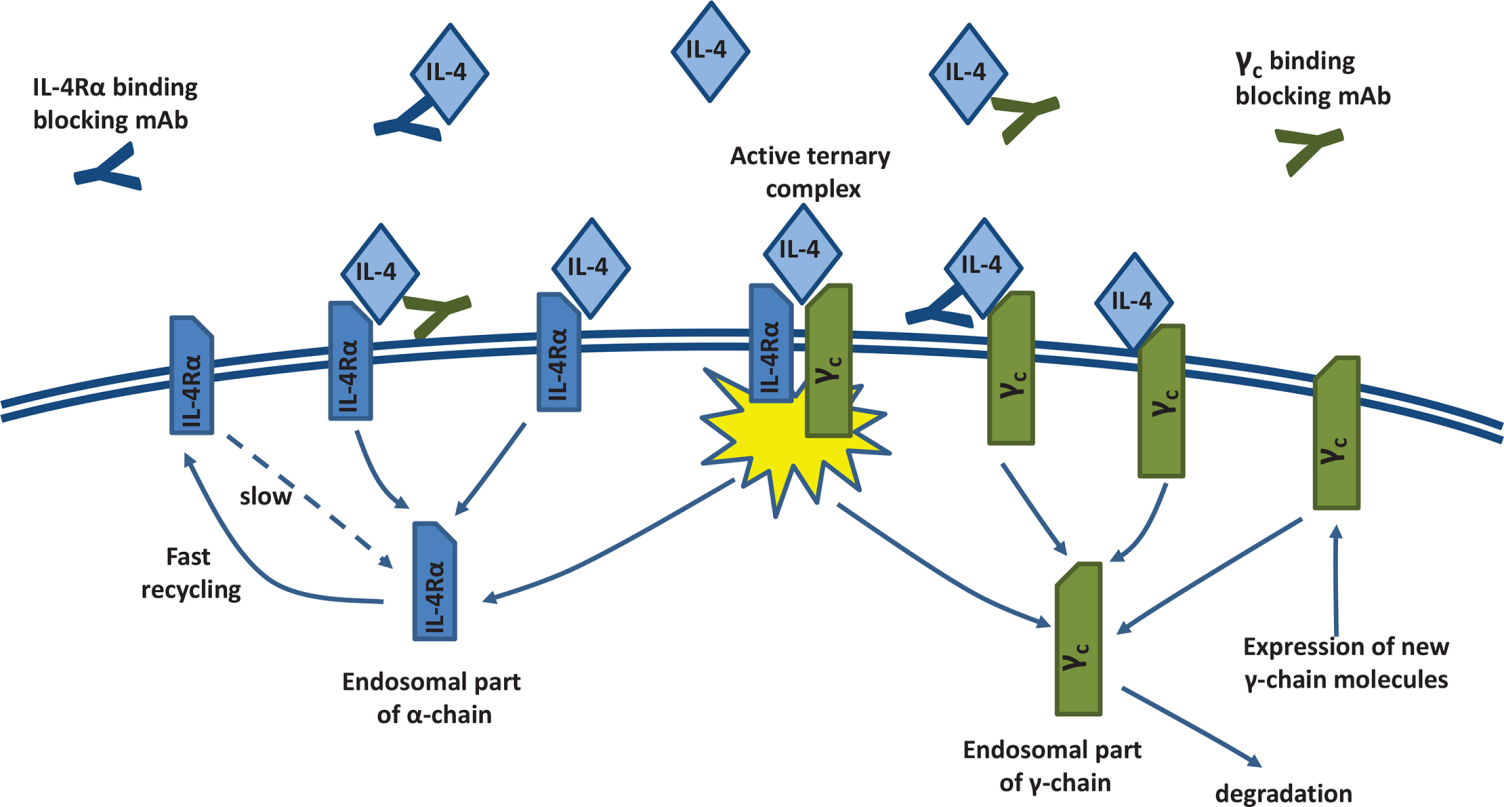
Cytokine-receptor model. The cytokine receptor consists of two subunits, the α-chain (IL-4Rα) and the common γ-chain (CD132). IL-4Rα subunits are internalized and recycled, while common γ-chain, antibody and cytokine are degraded after internalization.

The structure of the model involving a cytokine receptor with multiple subunits is shown in Fig 2. At the membrane surface of T cells, both IL-4Rα (approximately 1,000 molecules per cell) and γ_c_ subunits (approximately 6,000 molecules per cell) are present [29]. IL-4 molecules from the extracellular space preferentially bind first the high-affinity IL-4Rα subunit; this binary complex then meets a γ_c_ subunit within the two-dimensional cell membrane space, to form an active ternary complex (IL-4Rα/IL-4/γ_c_). Two types of antibodies may be considered, one which would block cytokine binding to its receptor *via* the α-chain, another one *via* the γ-chain.

Receptor subunits may next be internalized in an unbound state (at a relatively slow rate) or as bound to either a cytokine or to a cytokine complexed with a mAb that blocks binding to the other receptor chain (at a relatively faster internalization rate). As shown previously [30], the internalization rate is the same for binary (IL-4Rα/IL4) and ternary (IL-4Rα/IL-4/γ_c_) complexes. Receptor subunits may then be recycled to the cell surface (IL-4Rα) or be degraded within the endosomal space (γ_c_) [31–33]; cytokine and antibody, however, are degraded within the endosomal space.

Most model reactions are standard biochemical mass-balance equations. Model equations and model parameters are given in (S1 Appendix).

When a cytokine binds to both of its receptor subunits, it creates a signaling complex which stimulates T-cell proliferation, as shown experimentally [1, 5]. The proliferation rate (PR) dependence on the number of signaling complexes per cell was observed experimentally and can be described by an E_max_ function, as described by Fallon & Lauffenburger [25]:

$$PR=\frac{K_{pr}C}{C+{EC}_{50}}$$

where *K*_*pr*_ is the maximal proliferation rate, C is the number of signaling complexes per cell, and EC_50_ is the number of signaling complexes per cell at 50% of the maximal proliferation rate.

The increase in cell number over time is exponential and defined by the proliferation rate. This process is described by an equation that captures exponential growth:

$$\frac{dN_{cells}}{dt}=\frac{K_{pr}C}{\left(C+{EC}_{50}\right)}N_{cells}$$

The total amount of receptor subunits in the system also increases in time and proportionally to N_cells_.

## Results

### Agonistic effect driven by a cytokine-neutralizing antibody; a study via IL-4 / T-cell model simulations

IL-4 signaling enhancement agonistic effects occurring in the presence of an anti-IL-4 CNA or of a recombinant soluble receptor subunit (sIL-4Rα) have been reported *in vivo* [1] and *in vitro* [5]. More specifically, these agonistic effects occurred only within an intermediate range of mAb concentrations, yet were absent at smaller or larger ranges of mAb concentrations.

**Fig 3:**
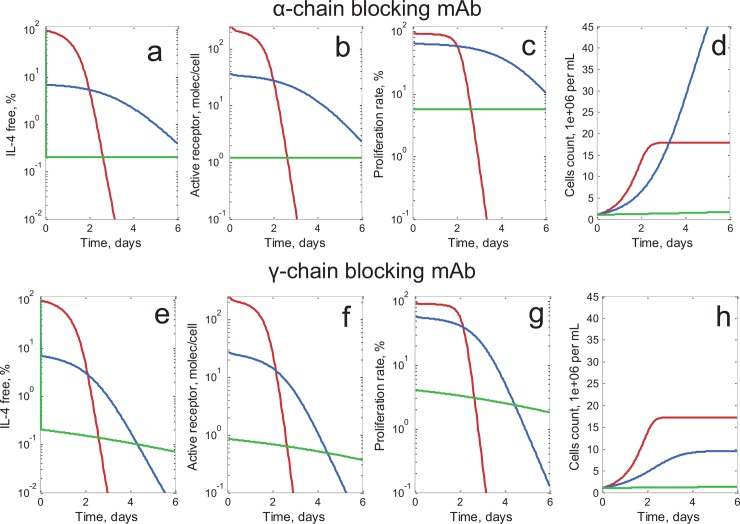
Kinetics of free interleukin, signaling, proliferation rate and cell count. In the model, T cells were incubated with IL-4 250 pM and different concentrations of mAb (5 pM—red lines, 250 pM–blue lines, 10000 pM–green lines; mAb K_D_ set at 20 pM) during 6 days. (a, e) Time course of free IL-4 (100% represents the initial IL-4 concentration of 250 pM); (b, f) Time course of number of the signaling complexes per cell; (c, g) Proliferation rates, expressed as an Emax function of the number of signaling complexes, with EC_50_ = 20 signaling complexes per cell; (d, h) Time course of cellular proliferation, expressed as T-cell count. The initial number of T cells is 1 x 10^6^ per ml.

The present modeling study is based on the *in vitro* experimental conditions set forth by Jung *et al*. [5]. Specifically, T cell (CT.h4S) proliferation was stimulated by the addition, to the cell medium, of 5 ng/ml (250 pM) of IL-4 and varying concentrations of sIL-4Rα. Since a soluble receptor sIL-4Rα and a mAb, preventing IL-4 binding to IL-4Rα on the cell surface, would represent the same mode of action, the mAb *vs*. the soluble receptor may be considered interchangeably. Model simulations were performed for various concentrations of both types of mAb (blocking binding to IL-4Rα or to γ_c_). The time courses of several model variables are shown in Fig 3.

First, we consider model simulations for a mAb that blocks IL-4 binding to IL-4Rα (Fig 3A–3D). At very low mAb concentrations, the cytokine binds to its receptor and rapidly gets internalized and cleared from the system (Fig 3A, red line). When the mAb is present at intermediate concentrations, it reduces the concentration of available free cytokine to a low, albeit sufficiently active level, thereby decreasing the elimination rate of the cytokine (Fig 3A, blue line). At high mAb concentrations, nearly all IL-4 molecules remain bound to antibodies over the entire incubation period; very low amounts of free cytokine would then be available (Fig 3A, green line). The amount of signaling complexes depends on the free IL-4 available to bind its receptor (Fig 3B). Time dependence of the proliferation rate is described by an E_max_ model function for the amount of signaling complexes (Fig 3C); these kinetics also are clearly related to the concentration of available free IL-4.

Proliferation, measured as numbers of T cells, is shown for varying mAb concentrations (Fig 3D). In the case of low mAb concentrations (red line), proliferation is higher in the earlier time period, as compared to intermediate mAb concentrations (blue line). After two days of incubation and with low mAb concentrations, most of the free IL-4 molecules will have been internalized: proliferation nearly stops; however, at intermediate mAb concentrations, proliferation persists, leading ultimately to a larger cell count. Therefore, identical initial concentrations of free IL-4 may produce significantly different cell proliferation counts over the long run; the proliferation effect may, in fact, be enhanced in the presence of intermediate *vs*. lower concentrations of the CNA, which “rescues” some of the initially free IL-4 molecules from rapid cellular internalization (lower initial “cellular consumption” of free IL-4).

Simulations for the same system, now with a mAb that blocks IL-4 binding to the γ-chain subunit, are next described (Fig 3E–3H). Cytokine binding with such a mAb does not prevent its target-mediated elimination from the system. Both free IL-4 molecules and IL-4/mAb complexes are able to bind IL-4Rα subunits at the cell membrane surface. The formation of these complexes (with or without mAb) accelerates the internalization of IL-4Rα subunits, as well as future cytokine degradation. As shown in Fig 3H, the γ-chain binding blocking mAb consistently inhibits T-cell proliferation in a dose-dependent manner.

A question remains, however: why would a mAb that blocks IL-4 binding to IL-4Rα effectively prevent the target-mediated elimination of IL-4? In principle, such a mAb/IL-4 complex would still be able to bind the γ-chain and therefore undergo cellular internalization as well. However, it turns out that the binding affinity of IL-4 to γ_c_ is rather weak (150 μM, as compared to the 0.6 nM affinity of IL-4 to the α-chain; a 100,000-fold difference indeed). Therefore, internalization of such small amounts of binary complex IL-4/γ_c_ (with or without mAb) may be considered as negligible.

**Fig 4:**
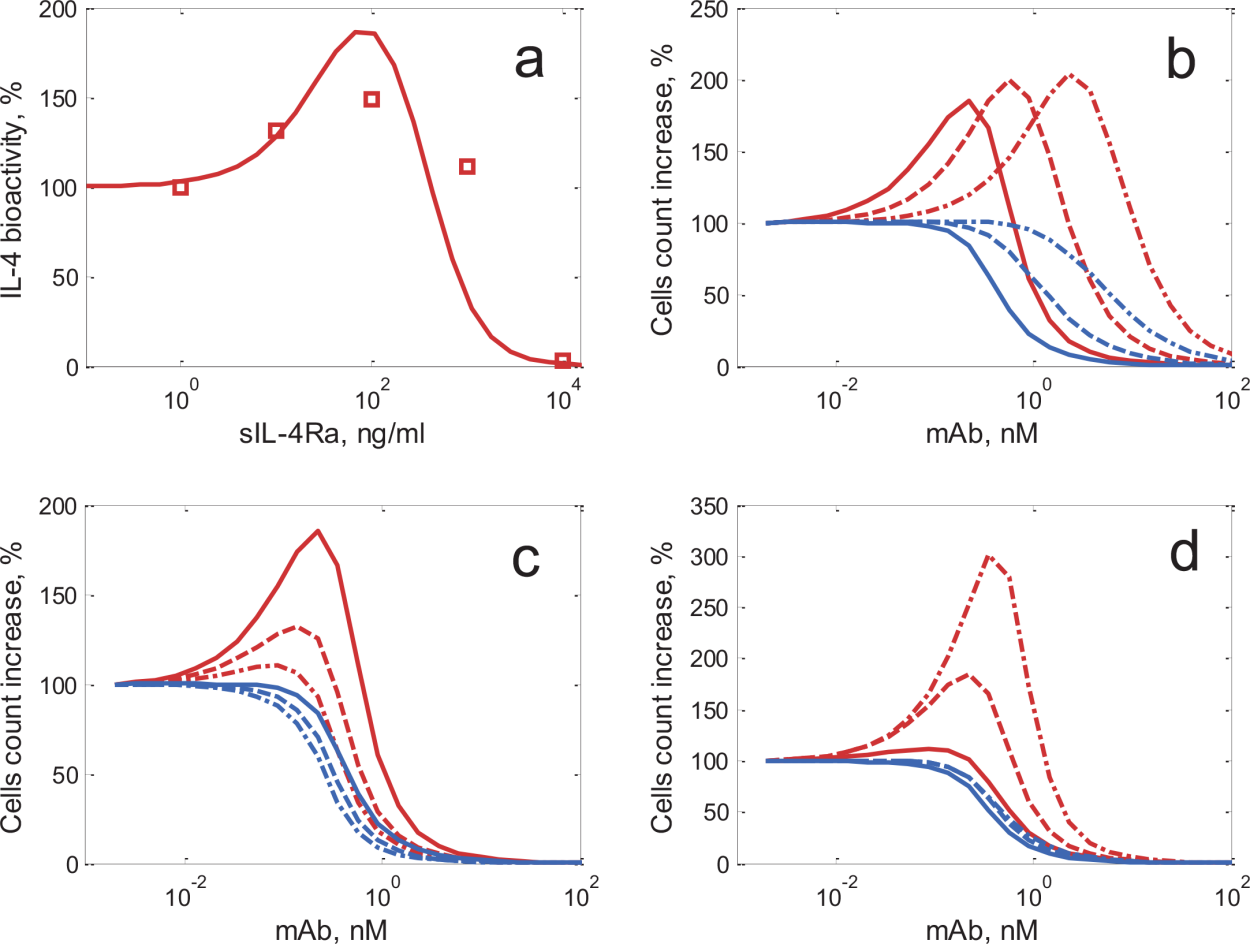
The agonistic effect in the IL-4/T cell model. Blue lines are for the γ-chain blocking antibody, red lines are for the α-chain blocking antibody. (a) Reproduction of the sIL-4Rα agonistic effect from [5]. IL-4 bioactivity measurements (red squares) were normalized to baseline (incubation with 250 pM IL-4, without sIL-4Rα). For sIL-4Rα /IL-4 binding, a K_D_ of 600 pM was used [29]; the initial number of T cells (2e+05 per ml) was estimated from the experimental description. (b,c,d) Dependencies of the agonistic effect upon parameter values in the IL-4/T cell model (sensitivity analysis), (b) Effect of varying IL-4/mAb binding affinities (solid, dashed and dashed-dotted lines are for K_d_ values of, respectively, 20, 100 and 500 pM); (c) Effect of varying EC_50_ values in the proliferation rate Emax function (solid, dashed, and dashed-&-dotted lines are for, respectively, 20, 50 and 100 signaling complexes per cell); (d) effect of varying incubation times (solid, dashed and dashed-dotted lines are for, respectively, cell counts measured after 2, 3, and 5 days). In all simulations, reference parameters were set as follows: initial IL-4 concentration was 250 pM and the initial number of T cells was 1e+06 per ml, other parameter values: IL-4/mAb binding K_D_ = 20 pM; EC_50_ = 20 signaling complexes per cell; incubation time = 3 days. 100% of the effect corresponds to the calculated number of cells for model parameters (K_D_, incubation time, EC_50_) set in the absence of any mAb. With an antibody that blocks the binding to the α-chain (red lines), the agonistic effect may occur. With an antibody that blocks the binding to the γ-chain, a monotonic decrease in the cytokine effect is observed (blue lines).

To explore the influence of model parameters on the occurrence of the agonistic effect, simulations were performed over a wide range of initial mAb concentrations, while keeping the initial IL-4 concentration fixed (Fig 4). As it shown in Fig 4A, the model adequately reproduces the agonistic effect described experimentally by Jung *et al*. [5]. In this experiment, T-cell proliferation effects of IL-4 (250 pM) with varying concentrations of sIL-4Rα were measured. The incubation period was 3 days, and IL-4 bioactivity was subsequently measured *via* [^3^H]thymidine incorporation. In the model, we assumed that [^3^H]-timidine incorporation into the DNA of proliferating T cells was proportional to the increase of T-cell counts during the last 6 hours of simulations: ΔN_cells_ = N_cells_(t = 78 h) ‒ N_cells_(t = 72 h). To then compare model-predicted simulations with experimental data, we normalized values to baseline (cell proliferation calculated without the addition of a mAb or sIL-4Rα).

Of further interest would be to explore the dependence of this model-predicted agonistic effect upon specific parameter values in the model, and as related to experimental conditions. We thus studied the agonistic effect as a function of varying mAb/IL-4 binding affinities. We next studied the dependency of the agonistic effect upon mAb concentrations. The concentration of mAb optimal for the agonistic effect was nearly proportional to K_D_ (Fig 4B). Since the EC_50_ value in the proliferation rate E_max_ function can differ across various types of T cells, we also tested the agonistic effect for varying EC_50_ values. Smaller EC_50_ values led to a stronger agonistic effect (Fig 4C). A minimal threshold number of signaling complexes was required to generate proliferation in these simulations; this threshold depended directly on the EC_50_ value in the model.

Finally, we tested dependency upon incubation times of T cells with IL-4 and mAb. As can be observed in Fig 4D, the agonistic effect (increase in effect above 100%) does not occur before the end of day 2 following the start of the experiment (red solid line); rather, the effect occurs and becomes more pronounced over time, at day 3 and day 5 (red dashed, and red dashed-&-dotted lines). This corresponds to Fig 3D, where, at day 2, the increase in antibody concentration from zero to maximum only leads to a monotonic decrease in cell proliferation (red line: minimum concentration; green line: maximum concentration). At day 5 (Fig 3D), however, when antibody concentration increases from a small amount (red line) to a higher value (blue line), proliferation increases, and the agonistic effect is observed; adding a CNA leads to an increase in proliferation rather than a decrease. With further increases in mAb concentrations (green line), proliferation decreases again. Therefore, the agonistic effect occurs only 2 to 3 days after the start of the experiment, hence a time delay for the occurrence of the effect is observed in these simulations, similarly to what has been observed experimentally.

## Discussion

### Agonistic effect: Summary of proposed mechanism

This modeling & simulation study, in accordance with existing experimental data, provided mechanistic insights into the agonistic effect, which arises under certain conditions in a triangular system involving a cytokine (interleukins used here as a case study), its receptor, and a cytokine-neutralizing antibody (CNA). Our simulations lend strong support to the following scenario: (1) at low (or zero) concentrations of the CNA, an interleukin may bind to its corresponding receptor, subsequently getting internalized and cleared from the system at a relatively fast rate. (2) However, for an optimal, intermediate range of CNA concentrations, interleukins bound to CNAs results in a pool or “depot” of interleukin-CNA complexes, which effectively reduces the concentration of free interleukin, at the same time also “rescues” free interleukins from a relatively fast internalization and clearance via intracellular endosomal degradation. Over time, the progressive internalization of free interleukin changes the equilibrium between concentrations of free interleukin *vs*. interleukin-CNA complex, which itself results in the gradual release of free cytokine (and sustained intracellular signaling over time) from the depot complex, and hence a possible enhanced agonistic effect in cell effector response, such as T-cell proliferation. (3) At larger CNA concentrations, most cytokine molecules are bound to CNAs; only a small number of free cytokine molecules remain available, over time, for further intracellular signaling.

Fig 3D illustrates how free IL-4 relates, over time, to a cell effector response such as proliferation: proliferation is initially high, given small amount of CNAs (red line) and as compared to intermediate amounts of CNAs (blue line); however, given sufficient time, most of the free IL-4 is all”consumed” and proliferation nearly stops, at lower CNA concentrations, while the initial slower proliferation (at equal initial free IL-4) is sustained over time and results in larger final cell counts, in the presence of intermediate levels of CNAs. This agonistic effect thus requires some time to build up (initial lag time observed), a feature which is also confirmed experimentally.

The proliferation rate depends on the number of interleukin/receptor signaling complexes and in a non-linear fashion, which can be adequately described using an E_max_-type function. Consistent with this E_max_ model, it has been suggested previously that an optimal concentration of free ligand (interleukin) would be tightly related to an optimal number of ligand/receptor signaling complexes to generate and sustain proliferation, while an excessive number of signaling complexes would only lead to an increase of endocytotic ligand degradation without further significant increase in proliferation.

### *In vivo* considerations

The agonistic effect, given certain CNA concentrations, has been observed in a number of *in vivo* experiments (see Table 1). Agonistic effects of a larger magnitude may actually be observed *in vivo*, as compared to the same agonistic effect *in vitro*. For example, Phelan *et al*. [8] performed injections in mice, with IL-4 and anti-IL-4 CNA mixtures at low (5:1) and high (5000:1) of CNA-to-cytokine weight ratios. An approximately up to 10-fold increase in CD8+ cell proliferation in spleen was measured 3 days following the injection, in mice which received the low CNA dose. However, no significant effect on CD8+ cell proliferation was observed in mice which received the high CNA dose.

The essential difference between the *in vitro* and *in vivo* settings described here, also applicable across cytokines, is the presence of an additional systemic clearance mechanism *in vivo*. Because most cytokines are relatively small proteins, they are effectively eliminated *via* renal clearance, thus exhibit short half-lives *in vivo*, which also limits their pharmacological applications. It is well known that fusion proteins (*e*.*g*., a cytokine linked to an IgG Fc fragment) exhibit a significantly reduced clearance, combined with a prolonged pharmacological action *in vivo*. Binding to a mAb molecule may thus prolong the half-life of the smaller molecular weight cytokine *in vivo*.

**Fig 5:**
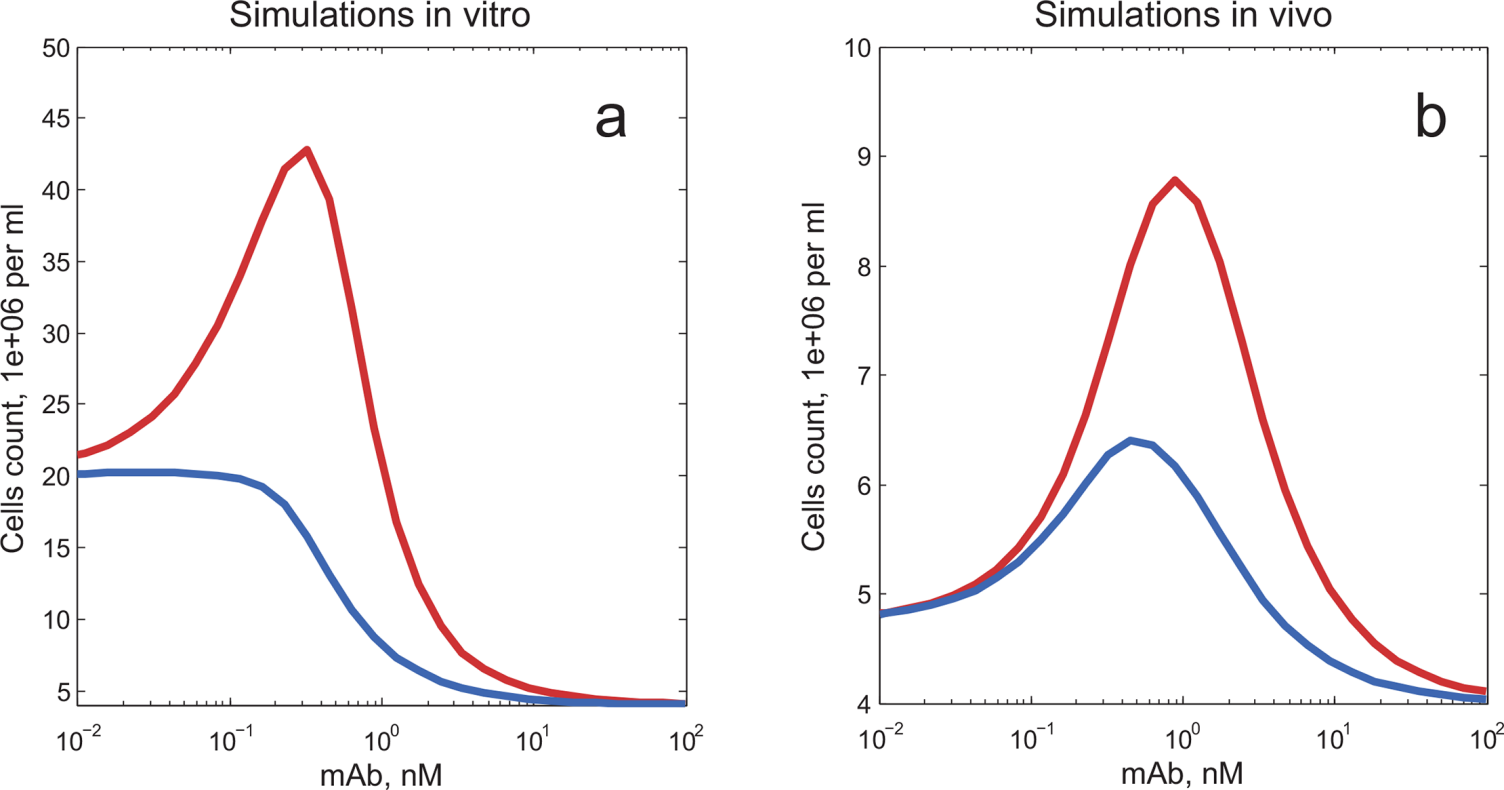
Agonistic effect of an IL-4 neutralizing mAb: model simulations for an *in vivo* case *vs*. an *in vitro* case. Red lines are for the α-chain blocking mAb, blue lines are for the γ-chain blocking mAb. (a) simulations for the *in vitro* case, (b) simulations for the *in vivo* case. The following experimental conditions were used in the model simulations: incubation time = 3 days; initial concentration of IL-4 = 250 pM; initial cells density = 4e+06 cells/ml; mAb/IL-4 binding K_D_ = 20 pM; EC_50_ = 20 signaling complexes per cell. For simulations of the *in vivo* conditions, systemic elimination rates of 1.26 1/h and 0.004 1/h were added, respectively, for IL-4 and the mAb.

To explore the effect of systemic clearance of a cytokine *in vivo*, we extended the model *via* the addition of a clearance mechanism for free IL-4 (relatively fast, renal), as well as a slow systemic clearance of both the antibody and the antibody/cytokine complex. Both types of anti-IL-4 CNAs (whether blocking binding to the α-chain or to the γ-chain) will in fact slow down renal clearance of IL-4 in comparable terms, but as derived from the properties of the cytokine-receptor interactions described in this paper, only the α-chain binding blocking CNA is capable of preventing target-mediated disposition of IL-4. In our simulations (Fig 5), we could indeed demonstrate larger relative increases in T-cell proliferation, in full agreement with the *in vivo* results reported by Phelan *et al*. [8].

In Fig 5, the base level (Y-axis) is taken to be equal to the initial cell count (4.0e+06 cells/ml). In the case of *in vitro* modeling (Fig 5A, red curve), both IL-4 alone and IL-4/α-chain blocking mAb complexes at optimal concentration lead to a large increase in cell count (up to 20e+06 cells/ml and up to 43e+06 cells/ml for, respectively, IL4 and IL4/mAb). The addition of the antibody amplifies the cell count increase caused by IL-4 alone, by a factor of 2.4.

In the case of *in vivo* modeling (Fig 5B), the addition of IL-4 alone increases the cell count only by 0.8e+06 cells/ml. The effect of IL-4 alone on T-cell proliferation is weak, due to a fast systemic elimination of free IL-4. The addition of an α-chain blocking mAb (red curve) at optimal concentration (about 1 nM) significantly potentiates proliferation (T-cell numbers increase by 4.8e+06 cells/ml at maximum value). The increase in cell count, as caused by mAb/IL4, is six folds larger than the cell count increase caused by IL-4 alone.

We thus conclude that the CNA agonistic effect (relative to the action of cytokine alone) may be even stronger *in vivo*, as compared to the same protagonists, concentrations and affinities *in vitro*. There is dual “free cytokine rescue” feature of the CNA *in vivo*: (a) as a cytokine depot, avoiding faster initial cytokine consumption via receptor-mediated internalization, (b) as a cytokine depot, avoiding faster renal clearance of the small molecule free cytokine. Evidently, other regulatory mechanisms may occur *in vivo* (*e*.*g*., immune cell interactions), thereby reducing or amplifying CNA-dependent cytokine signaling. Yet if such additional regulations do not significantly alter the agonistic effect, CNA-dependent slowdown of renal clearance is sufficient to describe the amplification of the agonistic effect *in vivo*.

### Other attempts to explain the CNA agonistic effect

In the review by Mostböck [9], experimental observations of the agonistic effect are reported for a variety of cytokines, including IL-2, IL-3, IL-4, IL-5, IL-6, IL-7, IL-15, TNF-α, for *in vitro* or *in vivo* conditions. CNAs and ligand/receptor systems also occur in healthy subjects as well as patients suffering of various disease conditions, including autoimmune diseases, infectious diseases, and various cancers [9]. It has also been noted that naturally occurring CNAs may serve as positive and negative self-regulatory mechanisms of cytokine signaling in the body [34].

Various attempts have been made previously, to explain the agonistic effect–yet a fundamental mechanistic understanding has been lacking. The various explanations proposed previously have called for a variety of mechanisms of action, and/or were specific to certain cytokines. They were also of a hypothetical nature, and conclusions about the actual mechanism(s) were still described as “elusive and enigmatic” [10,11].

This agonistic effect has been studied theoretically, for the *in vivo* situation, in [35]. In there, and similarly to the modeling results presented here, the importance of nonlinearity in cytokine/receptor binding was suggested. However, in the model presented in [35], the agonistic effect appears rather as the result of an increase in a cytokine’s half-life, via antibody binding, which would prevent fast clearance and would lead to an increase in apparent exposure to the cytokine (defined as time-averaged receptor occupancy). This model, however, cannot describe the agonistic effect occurring in an *in vitro* setting, where systemic clearance of the cytokine is absent. Finally, the simple pharmacokinetic model proposed in [35] cannot answer in details how the agonistic mechanism would arise.

Impact of IL-2 and IL-2/mAb therapies on T-cell dynamics were studied theoretically in [36, 37]. Authors considered potentiation of immunity, by blocking the IL-2R α-chain *via* a mAb. The proposed explanation of the agonistic effect was based on the role of IL-2 in the interplay between CD4+ helper and regulatory cells. However, such a mechanism would be IL-2 specific and based on interactions of different cell types. Therefore, it cannot explain the agonistic effect appearing *in vitro*, with other cytokines (Table 1), and where only one type of immune cells is in play in the system.

It has been suggested previously that an anti-IL-7 CNA, while binding IL-7, serves in fact as a depot for free interleukin, “rescuing” it from earlier and faster receptor-mediated consumption [11]. They further suggested that this effect of free cytokine rescuing via a depot may be a generic feature in other settings, not limited to cytokines, but applicable to other ligand-receptor interactions.

Such features and associated kinetic behavior are reproduced in our mathematical model. We show that the CNA acts as a depot for free interleukin, to then provide interleukin-dependent signaling (*via* free interleukin consumption) in a more evenly spread-out fashion over time. The occurrence of such an agonistic effect may be a generic feature within such ligand-receptor systems, since the application of the present model is not restricted to a particular cytokine and its receptor, and may indeed describe generic interactions between a ligand and its receptor.

In this paper, cell proliferation was taken as the effector response of choice, to “express” the agonistic effect. Other effector responses downstream of the ligand/receptor signaling may occur in such paradoxical agonistic fashion, as long as there is a non-linear relationship between the effector response under consideration versus the number of signaling complexes (a necessary condition for the agonistic effect to occur *in vitro*).

### Conclusions

The mechanism underlying the agonistic effect and described in this paper arises from basic properties of a rather generic cytokine-receptor system. It holds for a variety of cytokine/receptor systems (Table 1) and does not rely, for example, on a particular ‘signature’ of a cytokine. The range of concentrations and affinities of molecules would certainly differ across various ligand/receptor molecular species, for the agonistic effect to arise.

In particular, the most compelling experimental data have been reported by Zabeau *et al*. [14], where the bioactivity of human IL-5 in combination with varying concentrations of IL-5 neutralizing mAbs was explored *in vitro*. In these IL-5 experiments, the measured effect is strikingly similar to the effect demonstrated by the present model simulated for IL-4, see Section 2 in (S1 Appendix). This supports the view that this paradoxical agonistic effect, as observed in the presence of CNAs, is of a general nature for such cytokine-receptor systems, and may effectively arise, regardless of cytokine-specific aspects (*e*.*g*., IL-4 and IL-5 are from different cytokine families). Thus, our model can be tuned to reproduce a host of experimental observations made for these various cytokines.

Since cytokine/CNA complexes may produce stronger, or more sustained signaling *vs*. free cytokine alone, they may be exploited in pharmacological action and used for the purposes of increasing the apparent potency of a therapeutic drug and lowering therapeutic doses needed, in diseases where exogenous cytokines are used as therapeutics. Therapeutic applications of cytokine/CNA complex-dependent agonistic effects have been discussed in the context of various diseases. Therapeutic uses of IL-2/anti-IL-2 antibody complexes have been reviewed for cancer and autoimmune diseases [19], treatment of tumors [13], anti-bacterial and anti-viral infections [21, 38], multiple sclerosis [22], and transplantation tolerance [23]. Therapeutic uses for treatment of immunodeficiency and cancer of IL-7/anti-IL-7 antibody complexes have been discussed [15]. In many of these, increasing the apparent potency of a cytokine (through the use of the cytokine-CNA agonist effect) while reducing the actual administered dose of drug (cytokine) would also allow to better control for toxicity burden and adverse safety events.

The model proposed in this paper, together with an exploration of the agonistic mechanism may be exploited towards the design of therapeutic drug complexes of cytokine/CNA, dosing regimen optimization, and prediction of targeted immune cell responses.

## Supporting Information

S1 Appendix Model equations and parameters are given in Section 1 of the Appendix. Experimentally observed agonistic effect data for the IL-5 system confirm the agonistic effect featured in the model as shown in Section 2 of the Appendix.
(PDF)
