# Supplementary material for: A Generic Mechanism for Enhanced Cytokine Signaling via Cytokine-Neutralizing Antibodies
Source: PLoS One. 2016 Feb 12;11(2):e0149154. doi: 10.1371/journal.pone.0149154 (PMC4752257; doi:10.1371/journal.pone.0149154)
Supplement: S1 Appendix — Experimentally observed agonistic effect data for the IL-5 system confirm the agonistic effect featured in the model as shown in Section 2 of the Appendix. (PDF) [file pone.0149154.s001.pdf]

A Generic Mechanism for Enhanced Cytokine Signaling *via* Cytokine-Neutralizing Antibodies

Boris Shulgin, Gabriel Helmlinger and Yuri Kosinsky

Supplementary materials

Section 1. Model description

The equations provided here describe the kinetic reactions of the cytokine-receptor system shown in Fig. 2 (main text of our paper).

$$\frac{dIL}{dt} = -k_{on}^{mAb1} \cdot IL \cdot \ mAb_1 + k_{off}^{mAb1} \cdot IL\_mAb_1 - k_{on}^{mAb2} \cdot IL \cdot \ mAb_2 + k_{off}^{mAb2} \cdot IL\_mAb_2 - k_{on}^{IL\_\alpha} \cdot IL \cdot \ \alpha + k_{off}^{IL\_\alpha} \cdot IL\_\alpha - k_{on}^{IL\_\gamma} \cdot IL \cdot \ \gamma + k_{off}^{IL\_\gamma} \cdot IL\_\gamma$$

$$\frac{dmAb_1}{dt} = -k_{on}^{mAb1} \cdot IL \cdot \ mAb_1 + k_{off}^{mAb1} \cdot IL\_mAb_1$$

$$\frac{dmAb_2}{dt} = -k_{on}^{mAb2} \cdot IL \cdot \ m + k_{off}^{mAb2} \cdot IL\_mAb_2$$

$$\frac{dIL\_mAb_1}{dt} = k_{on}^{mAb1} \cdot IL \cdot \ mAb_1 - k_{off}^{mAb1} \cdot IL\_mAb_1 - k_{on}^{ILmAb1\_\gamma} \cdot IL\_mAb_1 \cdot \gamma + k_{off}^{ILmAb1\_\gamma} \cdot IL\_\_\gamma\_mAb_1$$

$$\frac{dIL\_mAb_2}{dt} = k_{on}^{mAb2} \cdot IL \cdot \ mAb_2 - k_{off}^{mAb2} \cdot IL\_mAb_2 - k_{on}^{ILmAb2\_\alpha} \cdot IL\_mAb_2 \cdot \alpha + k_{on}^{ILmAb2\_\alpha} \cdot IL\_\_\alpha\_mAb_2$$

$$\frac{d\alpha}{dt} = -k_{on}^{IL\_\alpha} \cdot IL \cdot \ \alpha + k_{off}^{IL\_\alpha} \cdot IL\_\alpha - k_{on}^{IL\gamma\_\alpha} \cdot IL\_\gamma \cdot \alpha_{dens} + k_{off}^{IL\gamma\_\alpha} \cdot IL\_\alpha\_\gamma - k_{int}^{\alpha} \cdot \alpha \ + k_{rec}^{\alpha} \cdot \alpha_{int} \ + k_{pr} \cdot C_{act}/(C_{act} + EC_{50}) \cdot \alpha_{tot}$$

$$\frac{d\gamma}{dt} = -k_{on}^{IL\_\gamma} \cdot IL \cdot \ \gamma + k_{off}^{IL\_\gamma} \cdot IL\_\gamma - k_{on}^{IL\alpha\_\gamma} \cdot IL\_\alpha \cdot \gamma_{dens} + k_{off}^{IL\alpha\_\gamma} \cdot IL\_\alpha\_\gamma - k_{int}^{\gamma} \cdot \gamma + k_{synt}^{\gamma} \cdot N_{cells}$$

$$\frac{dIL\_\alpha}{dt} = k_{on}^{IL\_\alpha} \cdot IL \cdot \ \alpha - k_{off}^{IL\_\alpha} \cdot IL\_\alpha - k_{on}^{IL\alpha\_\gamma} \cdot IL\_\alpha \cdot \gamma_{dens} + k_{off}^{IL\alpha\_\gamma} \cdot IL\_\alpha\_\gamma - k_{int}^{acc} \cdot IL\_\alpha$$

$$\frac{dIL\_\gamma}{dt} = k_{on}^{IL\_\gamma} \cdot IL \cdot \ \gamma - k_{off}^{IL\_\gamma} \cdot IL\_\gamma - k_{on}^{IL\gamma\_\alpha} \cdot IL\_\gamma \cdot \alpha_{dens} + k_{off}^{IL\gamma\_\alpha} \cdot IL\_\alpha\_\gamma - k_{int}^{\gamma} \cdot IL\_\gamma$$

$$\frac{dIL\_\alpha\_\gamma}{dt} = k_{on}^{IL\alpha\_\gamma} \cdot IL\_\alpha \cdot \gamma_{dens} - k_{off}^{IL\alpha\_\gamma} \cdot IL\_\alpha\_\gamma + k_{on}^{IL\gamma\_\alpha} \cdot IL\_\gamma \cdot \alpha_{dens} - k_{off}^{IL\gamma\_\alpha} \cdot IL\_\alpha\_\gamma - k_{int}^{acc} \cdot IL\_\alpha$$

$$\frac{dIL\_\alpha\_mAb_2}{dt} = k_{on}^{ILmAb2\_\alpha} \cdot IL\_mAb_2 \cdot \alpha - k_{on}^{ILmAb2\_\alpha} \cdot IL\_\alpha\_mAb_2 - k_{int}^{acc} \cdot IL\_\alpha\_mAb_2$$

$$\frac{dIL\_\gamma\_mAb_1}{dt} = k_{on}^{ILmAb1\_\gamma} \cdot IL\_mAb_1 \cdot \gamma - k_{off}^{ILmAb1\_\gamma} \cdot IL\_\_\gamma\_mAb_1 - k_{int}^{\gamma} \cdot IL\_\gamma\_mAb_1$$

$$\frac{d\alpha_{int}}{dt} = k_{int}^{\alpha} \cdot \alpha + k_{int}^{acc} \cdot IL\_\alpha + k_{int}^{acc} \cdot IL\_\alpha\_mAb_2 + k_{int}^{acc} \cdot IL\_\alpha\_\gamma - k_{rec}^{\alpha} \cdot \alpha_{int}$$

$$\frac{dN_{cells}}{dt} = k_{pr} \cdot C_{act}/(C_{act} + EC_{50}) \cdot N_{cells}$$

where IL, mAb_1, mAb_2, α, γ are concentrations in the medium (in pM) of, respectively, free interleukin, the α-subunit binding blocking mAb, the γ-subunit binding blocking mAb, free α-subunits, and free γ receptor subunits. IL_α, IL_γ, IL_mAb_1, IL_mAb_2 represent concentrations of interleukin bound to, respectively, the α-subunit, the γ -subunit, and antibodies of the two different types. IL_α_γ, $IL\_\alpha\_mAb_2$, $IL\_\gamma\_mAb_1$ represent concentrations of the interleukin bound to, respectively, both receptor subunits, mAb_2 and the α subunit, mAb_1 and the γ subunit. α_{int} is the concentration of internalized α-subunits. N_{cells} is the cell density measured in 10^9 cells per L of medium. The total concentration of α-subunits is:

$$\alpha_{tot} = \alpha + IL\_\alpha + IL\_\alpha\_\gamma + IL\_\alpha\_mAb_2 + \alpha\_int$$

α_{dens} and γ_{dens} are the densities of receptor subunits on the cell surface (molecules per µm^2), calculated as:

$$\alpha_{dens} = \alpha \cdot 10^{-12} \cdot N_A /(N_{cells} \cdot S_{membr}),$$
$$\gamma_{dens} = \gamma \cdot 10^{-12} \cdot N_A /(N_{cells} \cdot S_{membr})$$

where S_{membr} is the T cell surface area estimate and N_A is the Avogadro constant. The effect of the signaling complex upon the proliferation rate (PR) was described by an E_{max} function, in accordance with experimental findings [Ref. S1]:

$$PR = k_{pr} \cdot C_{act}/(C_{act} + EC_{50})$$

where k_{pr} is the maximal proliferation rate, $C_{act} = IL\_\gamma\_\alpha \cdot 10^{-12} \cdot N_A/N_{cells}$ is the number of signaling receptor complexes per cell, and EC_{50} is the number of signaling complexes per cell at 50% of the maximal proliferation rate.

The rate of γ-subunit synthesis (in pM/h per 10^9 cell in 1 L) was calculated from the γ-subunit degradation rate (k_{int}^{γ} , 1/h) and its expression level estimate (6,000 molecules per cell, [Ref. S2]) at steady-state:

$$k_{synt}^{\gamma} = (6{,}000 \cdot 10^9/N_A) \cdot 10^{-12} \cdot k_{int}^{\gamma}$$

When addressing the *in vivo* context, we added additional clearance reactions to the model, which are the last terms in the equations below:

$$\frac{dIL}{dt} = -k_{on}^{mAb1} \cdot IL \cdot mAb_1 + k_{off}^{mAb1} \cdot IL\_mAb_1 - k_{on}^{mAb2} \cdot IL \cdot mAb_2 + k_{off}^{mAb2} \cdot IL\_mAb_2 - k_{on}^{IL\_\alpha} \cdot IL \cdot \alpha + k_{off}^{IL\_\alpha} \cdot IL\_\alpha - k_{on}^{IL\_\gamma} \cdot IL \cdot \gamma + k_{off}^{IL\_\gamma} \cdot IL\_\gamma - k_{el}^{IL} \cdot IL$$

$$\frac{dmAb_1}{dt} = k_{on}^{mAb1} \cdot IL \cdot mAb_1 + k_{off}^{mAb1} \cdot IL\_mAb_1 - k_{el}^{mAb} \cdot mAb_1$$

$$\frac{dmAb_2}{dt} = k_{on}^{mAb2} \cdot IL \cdot\ m + k_{off}^{mAb2} \cdot IL\_mAb_2 - k_{el}^{mAb} \cdot mAb_2$$

$$\frac{dIL\_mAb_1}{dt} = k_{on}^{mAb1} \cdot IL \cdot\ mAb_1 - k_{off}^{mAb1} \cdot IL\_mAb_1 - k_{on}^{ILmAb1\_\gamma} \cdot IL\_mAb_1 \cdot \gamma + k_{off}^{ILmAb1\_\gamma} \cdot IL\_\_\gamma\_mAb_1 - k_{el}^{mAb} \cdot mAb_1$$

$$\frac{dIL\_mAb_2}{dt} = k_{on}^{Ab2} \cdot IL \cdot\ mAb_2 - k_{off}^{Ab2} \cdot IL\_mAb_2 - k_{on}^{ILAb2\_\alpha} \cdot IL\_mAb_2 \cdot \alpha + k_{on}^{ILAb2\_\alpha} \cdot IL\_\_\alpha\_mAb_2 - k_{el}^{mAb} \cdot mAb_2$$

Model parameter values corresponding to the IL-4 system are given in the Table S1.

Table S1. Parameter values used in the model

Reaction rates	Comments
IL binding with α-subunit	
$K_D^{IL\_\alpha} = 600\ pM$	[S2]
$k_{off}^{IL\_\alpha} = 10\ 1/h$	*
$k_{on}^{IL\_\alpha} = k_{off}^{IL\_\alpha} / K_D^{IL\_\alpha}$	
IL binding with γ-subunit	
$K_D^{IL\_\gamma} = \ 150\ \mu M = 150{,}000{,}000\ pM$	[S2]
$k_{off}^{IL\_\gamma} = 10\ 1/h$	*
$k_{on}^{IL\_\gamma} = k_{off}^{IL\_\gamma} / K_D^{IL\_\gamma}$	
Binding of IL_α complex with γ-subunit	
$K_D^{IL\alpha\_\gamma} = 1.0$ molec/µm^2	0.1 – 2.0 molecules/µm^2 [S2]
$k_{off}^{IL\alpha\_\gamma} == 10\ 1/h$	*
$k_{on}^{IL\alpha\_\gamma} = k_{off}^{IL\alpha\_\gamma} / K_D^{IL\alpha\_\gamma}$	
$S_{membr} = 600\ \mu m^2$	T-cell surface area estimate [S2]
Binding of IL_γ complex with α-subunit	
$K_D^{IL\gamma\_\alpha} = \ K_D^{IL\alpha\_\gamma} \cdot K_D^{IL\_\alpha} / K_D^{IL\_\gamma}$	
$k_{off}^{IL\gamma\_\alpha} = 10\ 1/h$	*
$k_{on}^{IL\gamma\_\alpha} = k_{off}^{IL\gamma\_\alpha} / K_D^{IL\gamma\_\alpha}$	
Binding of mAb to IL	

$K_D^{IL\_mAb} = 20\ pM$	10 - 1000 pM *(free parameter)*
$k_{off}^{IL\_mAb} = 10\ 1/h$	*
$k_{on}^{IL\_mAb} = k_{off}^{IL\_mAb}/K_D^{IL\_mAb}$	
Other parameters	
$k_{int}^{\alpha} = 0.1\ 1/h$	Very slow [S4]
$k_{int}^{acc} = 3.2\ 1/h$	Accelerated internalization rate for α-subunits bound with IL and IL_γ_α complexes [S5, S6]
$k_{int}^{\gamma} = 0.3$ *1/h*	Internalization and degradation of free γ-subunits and γ-subunits bound with IL [S4]
$k_{rec}^{\alpha} = 8.4$ *1/h*	Recycling rate of internalized α-subunits, assumed to be equal to the transferrin receptor recycling rate [S7]
$EC_{50} = 20$ signaling complexes per cell *(free parameter)*	Parameters in the T-cell proliferation rate function $k_{pr} \cdot C_{act}/(C_{act} + EC_{50})$<br>10-100 (free parameter)
$k_{pr} = 0.063$ 1/h	Maximal T-cell proliferation rate; a minimum time of a 10-hr cell cycle is assumed
***In vivo* systemic elimination of IL, mAb and their complexes**	
$K_{el\_IL} = 0.63/0.5\ \ (1/h)$	
$K_{el\_mAb} = 0.63/(6 \cdot 24)\ \ (1/\mathrm{h})$	Same elimination rate for the mAb and the mAb/IL complex is assumed
Receptor subunits per cell	
Rα	1,200 (1,000÷1,600 copies/cell [S2])
γc	6,000 (5,000÷8,500 copies/cell [S2])
Subunit density in T cell membrane	
Rα	2 molecules/μm^2
γc	10 molecules/μm^2<br>(T-cell membrane surface area was assumed to be 600 μm^2 [S2])
*For the value of k_{off} found in all binding reactions, a typical value of 10 1/h was used	

Section 2. Experimentally observed agonistic effect data for the IL-5 system confirm the agonistic effect featured in the model

The most compelling experimental data for the agonistic effect have been reported by Zabeau *et al*. [Ref. S3], where the bioactivity of human IL-5, in combination with varying concentrations of IL-5 neutralizing mAbs, was explored *in vitro*. In these IL-5 experiments, the measured effect is strikingly similar to the effect demonstrated by our model simulated for IL-4.

IL-4 and IL-5 belong to different cytokine classes: IL-5 is a homodimer, whereas IL-4 is a monomer. However, essential properties relevant to the present modeling study are preserved in both systems: the interleukin binds to a specific chain with a high affinity (*e.g.*, IL-5 to IL-5Rα and IL-4 to IL-4Rα); and the binary complex of cytokine, with its α-chain, meets the common chain in the two-dimensional membrane space (the common β-chain for IL-5, and the common γ-chain for IL-4). It has been established [Ref. S3] that the anti-IL5 mAb 5A5 epitope overlaps with the IL-5Rα chain binding site. The anti-IL-5 mAb 1E1 binding, on the other hand, overlaps with the common β-chain interacting residues.

Our model reproduces CNA dose-dependent effects, similar to experimental data observed for IL-5, and under the experimental conditions found in Zabeau *et al*. [Ref. S3]. It demonstrates that the mechanism of the paradoxical agonistic effect may arise from basic properties of such cytokine-receptor systems. Therefore, our model is of a generic nature and can be tuned to describe agonistic effects for different cytokines.

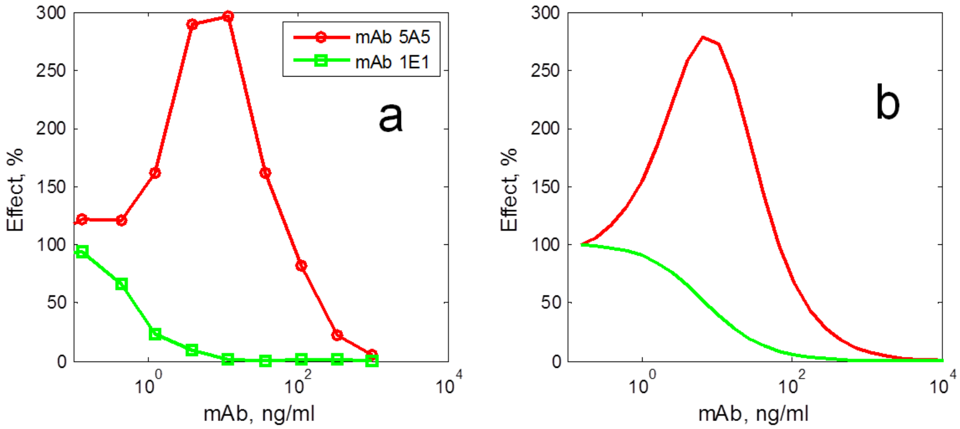

Figure S1. Experimentally observed agonistic effect for IL-5 is in agreement with the agonistic effect observed in our model.

Green lines are for common β-chain (a) and γ-chain (b) blocking antibodies, red lines are for the high-affinity α-chain blocking antibody. (a) *In vitro* experimental data from Zabeau *et al*. [Ref. S3]. (b) Agonistic effect from

our model simulations, using experimental conditions from the IL-5 study. The agonistic effect is materialized by a >100% effect – red curves – within an optimal range of antibody concentrations.

References

S1. Fallon EM, Lauffenburger DA. Computational Model for Effects of Ligand/Receptor Binding Properties on Interleukin-2 Trafficking Dynamics and T Cell Proliferation Response. Biotechnol. Prog. 2000; 16: p. 905-916.

S2. Whitty A, Raskin N, Olson DL, Borysenko CW, Ambrose CM, Benjamin CD, et al. Interaction affinity between cytokine receptor components on the cell surface. Proc. Natl. Acad. Sci. 1998; 95: p. 13165-70.

S3. Zabeau L, Van der Heyden J, Broekaert D, Verhee A, Vandekerckhove WSJ, Chalken I, et al. Neutralizing monoclonal antibodies can potentiate IL-5 signaling. Eur. J. Immunol. 2001; 31: p. 1087–1097.

S4. Morelon E, Dautry-Varsat A. Endocytosis of the Common Cytokine Receptor gamma Chain, Identification of sequences involved in internalization and degradation. 1998; 273(34): p. 22044-22051.

S5. Galizzi JP, Zuber CE, Cabrillat H, Djossou O, Banchereau J. Internalization of human interleukin 4 and Transient Down-regulation of Its Receptor in the CD23-inducible Jijoye Cells. 1989; 264(12): p. 6984-6989.

S6. Friedrich K, Kammer W, Erhardt I, Brändlein S, Arnold S, Sebald W. The two subunits of the interleukin-4 receptor mediate independent and distinct patterns of ligand endocytosis. Eur. J. Biochem. 1999; 265: p. 457-65.

S7. Ciechanover A, Schwartz AL, Dautry-Varsat A, Lodish HF. Kinetics of internalization and recycling of Transferrin and the Transferrin Receptor in Human Hepatoma Cell Line. 1983; 258(16): p. 9681-9689.
